# Nurse staffing and patient outcomes: a longitudinal study on trend and seasonality

**DOI:** 10.1186/s12912-016-0181-3

**Published:** 2016-10-14

**Authors:** Jianghua He, Vincent S. Staggs, Sandra Bergquist-Beringer, Nancy Dunton

**Affiliations:** 1 Department of Biostatistics, University of Kansas Medical Center, MS 1026, 3901 Rainbow BLVD, Kansas City, KS 66160 USA; 2 Health Services & Outcomes Research, Children’s Mercy Hospitals & Clinics, 2401 Gillham Rd, Kansas City, MO 64108 USA; 3 School of Nursing, University of Kansas Medical Center, 3901 Rainbow BLVD, Kansas City, KS 66160 USA

**Keywords:** Inpatient falls, Hospital-acquired pressure ulcers, Patient safety, Within-unit variability, Data aggregation

## Abstract

**Background:**

Time trends and seasonal patterns have been observed in nurse staffing and nursing-sensitive patient outcomes in recent years. It is unknown whether these changes were associated.

**Methods:**

Quarterly unit-level nursing data in 2004–2012 were extracted from the National Database of Nursing Quality Indicators® (NDNQI®). Units were divided into groups based on patterns of missing data. All variables were aggregated across units within these groups and analyses were conducted at the group level. Patient outcomes included rates of inpatient falls and hospital-acquired pressure ulcers. Staffing variables included total nursing hours per patient days (HPPD) and percent of nursing hours provided by registered nurses (RN skill-mix). Weighted linear mixed models were used to examine the associations between nurse staffing and patient outcomes at trend and seasonal levels.

**Results:**

At trend level, both staffing variables were inversely associated with all outcomes (*p* < 0.001); at seasonal level, total HPPD was inversely associated (higher staffing related to lower event rate) with all outcomes (*p* < 0.001) while RN skill-mix was positively associated (higher staffing related to higher event rate) with fall rate (*p* < 0.001) and pressure ulcer rate (*p* = 0.03). It was found that total HPPD tended to be lower and RN skill-mix tended to be higher in Quarter 1 (January-March) when falls and pressure ulcers were more likely to happen.

**Conclusions:**

By aggregating data across units we were able to detect associations between nurse staffing and patient outcomes at both trend and seasonal levels. More rigorous research is needed to study the underlying mechanism of these associations.

**Electronic supplementary material:**

The online version of this article (doi:10.1186/s12912-016-0181-3) contains supplementary material, which is available to authorized users.

## Background

In a 2002 national survey of physicians and the public, nurse understaffing was ranked as one of the greatest threats to patient safety in hospitals within the United States (US) [1]. According to the American Nurses Association (ANA), “when health care employers fail to recognize the association between RN staffing and patient outcomes, laws and regulations become necessary” [2]. In 2003 California mandated minimum registered nurse (RN)-to-patient ratios for hospitals to be met by January 1, 2004 [3]. Twelve other states subsequently issued staffing laws requiring hospitals to have either staffing committees responsible for plans and staffing policy or some form of disclosure and/or public reporting of staffing levels [2]. Perhaps due in part to policy changes, including the 2008 Centers for Medicare & Medicaid Services (CMS) rule change ending reimbursement for costs of certain hospital-acquired conditions, as well as to ongoing concerns with quality improvement, nurse staffing levels in US hospitals have increased substantially in recent years. From 2004 to 2011, total nursing hours per patient day (HPPD) on general care units of US hospitals increased by 11.5 % and registered nurse hours per patient day (RN HPPD) increased by 22.9 % [4].

It may be intuitive that more nurses can provide better patient care, but research findings about the association between nursing staffing and patient outcome have been inconclusive. The staffing law in California has been in effect for more than 10 years, but researchers did not find evidence of quality improvement associated with the legislation [5, 6]. A meta-analysis based on findings from a systematic review of the literature identified a consistent relationship between higher nurse staffing and lower patient mortality; however, findings regarding the association between nurse staffing and other outcomes such as falls, pressure ulcers, and urinary tract infections varied across studies, and overall results were inconclusive in a pooled analysis [7]. Recent studies provide little empirical evidence to clarify this finding. For pressure ulcers, Park et al. showed that higher RN HPPD was associated with lower unit-acquired pressure ulcer rates in adult care units [8], whereas other researchers reported that staffing and hospital-acquired pressure ulcers were not meaningfully associated [9] or they were associated in the opposite direction, that is, the higher staffing the higher pressure ulcer rate [10–12]. Other studies have also reported associations between higher nurse staffing and higher risk of adverse patient outcomes [13–15]. Most of these studies were based on cross-sectional analyses and some researchers considered the counter-intuitive findings as the result of inadequate risk adjustment [10, 15].

Examining the association of nurse staffing and patient outcomes from a longitudinal perspective may provide new information. According to some longitudinal studies and national surveys, rates of falls and hospital-acquired pressure ulcers in US hospitals decreased significantly in recent years [9, 16–18]. As nurse staffing levels in the US have increased during the same time period [4], it is expected that nurse staffing and rates of these nursing sensitive outcomes were inversely associated at trend level. At seasonal level, the rate of hospital-acquired pressure ulcers was found to be the highest in Quarter 1 of a year; the researchers hypothesized that the seasonality (seasonal pattern) in pressure ulcer rate was related to decreased staffing level in Quarter 1 attributable to patient volume [11]. When patient outcomes are sensitive to nurse staffing, we would expect that they are associated not only at trend level but also at seasonal level.

This study was designed to examine the longitudinal association between nurse staffing and patient outcomes, such as falls and hospital-acquired pressure ulcers, using data from the National Database of Nursing Quality Indicators® (NDNQI®). Our hypothesis was that nurse staffing and rates of these two outcomes were associated inversely at both trend and seasonal levels.

## Methods

### Data

This longitudinal study was based on 2004–2012 data from the NDNQI. Participating NDNQI hospitals submit unit-level nurse staffing and inpatient falls data monthly and pressure ulcers data quarterly. For this study, monthly nurse staffing and inpatient falls were collapsed into quarterly measures. Total inpatient falls include both injurious and non-injurious falls. Pressure ulcers in this study were limited to those that occurred after admission (hospital-acquired pressure ulcers), including stage I-IV ulcers and those that could not be staged. Detailed definitions of these outcomes and how they are measured were previously published [19–21]. Only adult critical care, step-down, medical, surgical, medical-surgical, and rehabilitation units were included in the analyses as these represent the vast majority of units that reported data to the NDNQI during 2004–2012. For each unit, only years with all four quarters of data on an outcome were considered for analyses. Units with at least 1 year of data were included in final analyses for each outcome. In total 13,339 units from 1622 hospitals met this inclusion criterion for inpatients falls and 12,435 units from 1527 hospitals met the inclusion criterion for pressure ulcers.

### Unit-level trend and seasonality

Decreasing trends in falls and pressure ulcers as well as seasonality in pressure ulcers have been reported in previous studies based on unit-level analyses using hierarchical generalized linear models [11, 17]. When we used a similar approach to examine the associations of changes in nurse staffing and changes in fall and pressure ulcer rates, inverse associations in trends were found but no seasonal associations were found. We hypothesized that seasonal association (signal) for these outcomes was undetectable because the within-unit variations (noise) were too large.

A preliminary analysis on the within-unit trend and seasonality of fall and pressure rates was conducted. There were 1240 units with complete fall data (36 quarters in 2004–2012). For each of these units, a linear model was used to test the trend (time) and seasonality (three dummy variables indicating four quarters) in the fall rate based on the 36 quarterly observations. The proportions of units with significant trend and significant seasonality among the 1240 units were calculated. A similar analysis was conducted for the pressure ulcer rate based on 848 units with complete pressure ulcer data.

### Grouping and aggregation

Our primary analyses were done based on variables aggregated over units within groups. We adopted this aggregation strategy to reduce random within-unit temporal variance (random variability in a unit’s outcomes from quarter to quarter) in the hope of capturing both trend and seasonal associations between staffing and outcomes. So units were divided into groups based on their data structures and all variables were aggregated across units within groups then the analyses were conducted at group level.

A grouping mechanism was developed to include all units in analyses. Less than 10 % of units had complete data in 2004–2012 for both falls (1240, 9.3 %)) and pressure ulcers (848, 6.8 %). Units had data available at different time points due to various reasons. Some hospitals dropped out of the NDNQI while other hospitals joined the NDNQI voluntarily during the study period. Sometimes units missed the deadlines of data reporting or failed to report for some other reasons, which created gaps in the longitudinal data. We created 9 indicator variables, one for each year of the study (2004–2012), to indicate whether each unit reported complete data for the study year or not. When we grouped units with identical values on these indicator variables, 345 unique groups for falls and 356 unique groups for pressure ulcers were identified. These groups were of various sizes: the largest ones had more than 1000 units while the smallest ones comprised a single unit; most groups had less than 100 units. By dropping some units’ data for certain years, small groups (<100 units) were merged into larger groups so that each final group had at least 100 units. For the analyses on falls, 42 final groups were built based on a total of 217,592 unit-level quarterly observations; for the analyses on pressure ulcers, 41 groups were built based on a total of 187,368 unit-level quarter observations (see Additional file 1: Figure S1, flowcharts of data preparation).

All variables were aggregated over units within groups at each quarter. To determine the fall rate, the total numbers of falls and patients days were summed over units within groups first, then the quarterly rates of falls per 1000 patient days were calculated for each group. To determine the pressure ulcer rate, the total numbers of patients with pressure ulcers and patients assessed for pressure ulcers were summed over units within groups first, then the quarterly proportions of patients with pressure ulcers were calculated for each group. Staffing variables were determined in a similar way.

### Variables

All outcome and staffing variables used for analysis were change scores from the first quarter with data in this study for each group (baseline). The outcome variables were changes in fall rate (the number of falls per 1000 patient days) and changes in pressure ulcer rate (the proportion of patients with hospital-acquired pressure ulcers) from baseline. Changes in rates of injurious falls and pressure ulcers of stage III or above (i.e., excluding stages I/II) were also analyzed as these outcomes are more costly. Staffing variables included change scores (from baseline) of total nursing hours per patient day (total HPPD) and percent of nursing hours provided by registered nurses (RN skill-mix).

To separate the associations between staffing and patient outcomes at the trend level from those at the seasonal level, each of the staffing variables was decomposed into two components: the annual mean and the quarterly difference from the annual mean. Computational formulas are shown below.

Change from baseline:

$$ HPPD\_Ch{g}_{ij}=HPP{D}_{ij}-HPP{D}_{11} $$

Annual mean:

$$ HPPD\_Ch{g}_{i.}=\left( HPPD\_Ch{g}_{i1}+ HPPD\_Ch{g}_{i2}+ HPPD\_Ch{g}_{i3}+PPD\_Ch{g}_{i4}\right)/4 $$

Seasonal change:

$$ \varDelta HPPD\_Ch{g}_{ij}= HPPD\_Ch{g}_{ij} - HPPD\_Ch{g}_{i.} $$

Decomposition:

$$ HPPD\_Ch{g}_{ij}= HPPD\_Ch{g}_{i.}+\varDelta HPPD\_Ch{g}_{ij} $$

where *i* = 1, 2, …, 9, *j* = 1, 2, 3, 4.

With this decomposition, the associations at the trend level can be captured by the coefficients of the annual means (*HPPD*_*Chg*_*i*._ and *RN*_*Skillmix*_*Chg*_*i*._), and the seasonal associations can be captured by the coefficients of the seasonal changes (*ΔHPPD*_*Chg*_*ij*_ and *ΔRN*_*Skillmix*_*Chg*_*ij*_).

Unit type and hospital characteristics were not considered in the analyses as these characteristics were inseparable after aggregation. Also, there was no control for patient risk, however, we would expect risk to average out to a large extent in aggregation. The analyses were designed to examine temporal associations between nurse staffing and patient outcomes in aggregate, not at hospital or unit level.

Weighted linear mixed models were used for group-level analysis. Linear mixed models with AR(1) correlation structure were chosen to accommodate the correlation among the repeated measures of patient outcomes within group. Weighted linear mixed models are linear mixed models with different weights applied to different subjects. For this study, the weight assigned to each group was its size (ie, number of units) so that groups with more units carried more weights in the models. All the analyses were conducted with STATA 13.1 SE (StataCorp, College Station, Texas, USA). Stata package *mixed* was used for modeling, and weighting was fulfilled with option *pweight (group size)* available for STATA 12 and later versions. The significance level for all tests was α = 0.05.

## Results

Table 1 shows hospital and unit characteristics at baseline for all units included in the final analyses of falls and pressure ulcers. The characteristics of the samples for falls and pressure ulcers were similar. Note that baselines for different groups could be in different calendar years as groups were determined by the data reporting pattern.

**Table 1:** Summary statistics for falls and hospital acquired pressure ulcers data at baseline

	Falls	Pressure Ulcers
Hospital^a^	*N* = 1622	*N* = 1527
Teaching	695 (42.9 %)	677 (43.9 %)
Magnet	119 (7.34 %)	132 (8.64 %)
Beds ≥300	438 (26.9 %)	431 (28.2 %)
Unit^b^	*N* = 13,339	*N* = 12,435
Critical Care	2837 (21.3 %)	2707 (21.8 %)
Step-Down	2021 (15.2 %)	1893 (15.2 %)
Medical	2517 (18.9 %)	2333 (18.8 %)
Surgical	1853 (13.9 %)	1718 (13.8 %)
Medical-Surgical	3452 (25.9 %)	3202 (25.8 %)
Rehab	659 (4.9 %)	582 (4.7 %)
RN HPPD	7.6 (4.3)	7.6 (4.3)
Non RN HPPD	2.7 (1.3)	2.6 (1.3)
Total HPPD	10.3 (4.0)	10.3 (4.0)
RN skill-mix	0.7 (0.2)	0.8 (0.2)
Total falls/1000 patient days	3.3 (2.7)	NA
Injurious falls/1000 patient days	0.9 (1.1)	NA
Hospital acquired pressure ulcer rate (%)	NA	6.9 (10.2)
hospital acquired pressure ulcer (Stage III or above) rate	NA	1.2 (3.8)
Group^b^	*N* = 42	*N* = 41
Size (number of units)	182 (111, 1240)	180 (101, 1222)
Number of quarterly observations/group	12 (4, 36)	12 (4, 36)

^a,b^All variables reflect baseline values. Summary statistics are mean (standard deviation) for continuous variable and count (%) for categorical variables

^c^Summary statistics are median (minimum, maximum)

### Unit-level trend and seasonality

**Fig. 1:**
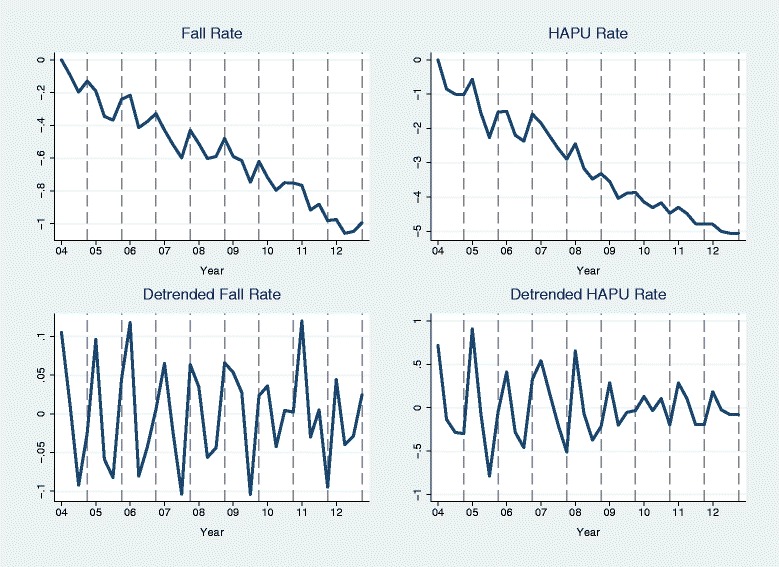
Aggregated quarterly fall rates and pressure ulcer rates. Quarterly fall rates were aggregated over 1240 units with all 36 quarters of fall data in 2004–2012 and pressure ulcer rates were aggregated over 848 units with all 36 quarters of pressure ulcer data. *Top plots* are change scores from Quarter 1 2004 and *bottom plots* are the change scores with the trend removed (minus annual means)

Seasonality in fall rate and pressure ulcer rate was observable at the group level but was not detectable in our preliminary analyses of single units. Figure 1 shows the change in fall rate and pressure ulcer rate aggregated over units with complete data (36 quarters), as well as these rates with annual trends removed. A seasonal pattern can be seen for both outcomes (Fig. 1 upper plots); both rates tend to be the highest in Quarter 1 or Quarter 4 and lowest in Quarter 2 or Quarter 3. At the single unit level, a statistically significant time trend in fall rate (Fig. 1 lower left plot) was found in 32.26 % (400/1240) of units but statistically significant seasonality was found in only 4.52 % (56/1240) of units. For the pressure ulcer rate (Fig. 1 lower right plot), a significant time trend was found in 37.50 % (318/848) of units but significant seasonality was found in only 3.66 % (31/848) of units. Because significant seasonality was expected to be found by chance in 5 % of the units when there was no seasonality at all (alpha = 0.05), test result suggests that there was no detectable seasonality in either outcome at the single unit level.

### Group-level trend and seasonality

**Fig. 2:**
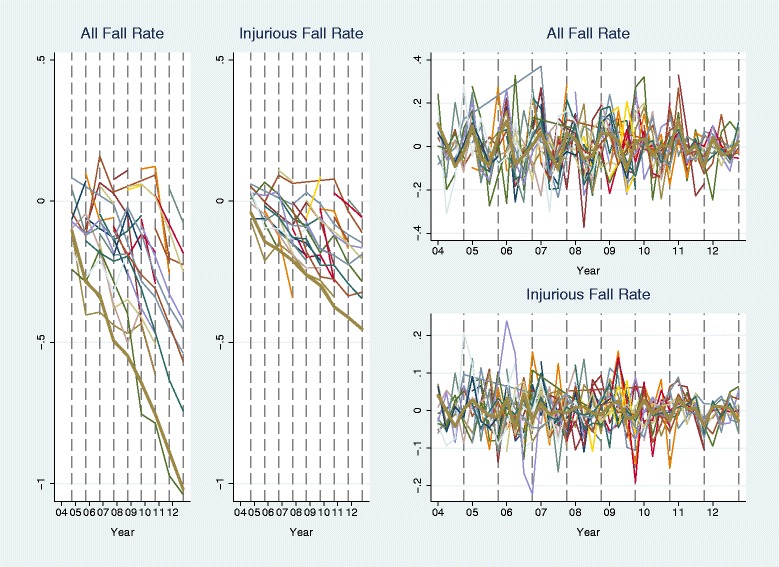
Trend and seasonal variation in fall rates. Trend is represented by the trajectory of annual mean rate and seasonal variation is represented by the trajectory of (quarterly rate – annual mean rate); rates were aggregated over units within 42 groups. The *thick line* in each plot is for the group of 1240 units with all 36 quarters of data in 2004–2012. Each *vertical dash line* denotes the fourth quarter of a year

**Fig. 3:**
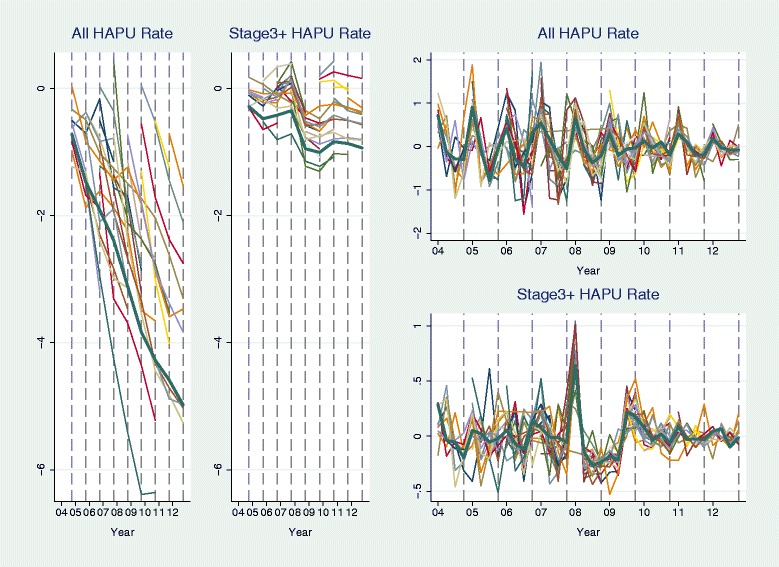
Trend and seasonal variation in rates of hospital-acquired pressure ulcers (HAPUs). Trend is represented by the trajectory of annual mean rate and seasonal variation is represented by the trajectory of (quarterly rate – annual mean rate); rates were aggregated over units within 41 groups. The *thick line* in each plot is for the group of 848 units with all 36 quarters of data in 2004–2012. Each *vertical dash line* denotes the fourth quarter of a year

Figures 2 and 3 show the group-level fall and pressure ulcer rates separated into annual means and seasonal variations (see Methods section for the decomposition method). The decreasing trends are strong and consistent across groups for both falls (Fig. 2: two plots on the left) and pressure ulcer rates (Fig. 3: two plots on the left). The seasonal pattern is more consistent across groups for pressure ulcer rates (Fig. 3: two plots on the right) than for fall rates (Fig. 2: two plots on the right). It seems that pressure ulcers were most likely to occur in Quarter 1 in a year and falls were more likely to occur in Quarter 1 and Quarter 4. For injurious falls, no seasonality is observable in the figure.

### Weighted linear mixed models

Table 2 shows the results of weighted linear mixed models using changes in total HPPD and RN skill-mix as covariates to model changes in fall and pressure ulcer rates. In terms of time trend, total HPPD was inversely associated (coefficient < 0) with all four adverse outcomes (*p* < 0.001); RN skill-mix was inversely associated with most outcomes (*p* < 0.001) except for the rate of pressure ulcers of stage III or above (*p* = 0.11). In terms of seasonality, total HPPD was inversely associated with all outcomes (*p* < 0.001); RN skill-mix was positively associated with total fall rate (*p* < 0.001) and total pressure ulcer rate (*p* = 0.03), inversely associated with the rate of pressure ulcers of stage III or above (*p* < 0.001), and not associated with injurious fall rate (*p* = 0.08).

**Table 2:** Weighted linear mixed models for falls and hospital acquired pressure ulcer rates^a^

Inpatient Falls				
Variables	Total Falls		Injurious Falls	
	Coefficient (95 % CI)	*P*-value	Coefficient (95 % CI)	*P*-value
Trend–annual mean				
Change in total HPPD	−0.36 (−0.51, −0.21)	<0.001	−0.12 (−0.17, −0.07)	<0.001
Change in RN skill-mix (1 percentage point)	−0.05 (−0.07, −0. 032)	<0.001	−0.04 (−0.05, −0.03)	<0.001
Seasonal variation				
Change in total HPPD	−0.18 (−0.24, −0.11)	<0.001	−0.06 (−0.09, −0.03)	<0.001
Change in RN skill-mix (1 percentage point)	0.07 (0.05, 0.10)	<0.001	−0.01 (−0.02, 0.00)	0.08
Hospital Acquired Pressure Ulcers				
Variables	All		Stage III or Above	
	Coefficient (95 % CI)	*P*-value	Coefficient (95 % CI)	*P*-value
Trend–annual mean				
Change in total HPPD	−1.82 (−2.56, −1.07)	<0.001	−0.49 (−0.71, −0.26)	<0.001
Change in RN skill-mix (1 percentage point)	−0.6 (−0. 20, −0.16)	<0.001	−0.38 (−0.84, 0.86)	0.110
Seasonal variation				
Change in total HPPD	−1.80 (−2.02, −1.59)	<0.001	−0.12 (−0.17, −0.07)	<0.001
Change in RN skill-mix (1 percentage point)	0.11 (0.01, 0.20)	0.03	−0.04 (−0.05, −0.03)	<0.001

^a^Intercepts and the estimated autocorrelation coefficients of autoregressive models of order 1 (AR1) are not shown in this table

Based on aggregated data, highly significant associations between nurse staffing and patient outcomes were found at both trend and seasonal levels. At trend level, increases in total nurse staffing and increases in the proportion of RN staffing were both associated with decreases in falls and pressure ulcers. At seasonal level, increases in total nurse staffing remained associated with decreases in falls and pressure ulcers, but findings were mixed for the proportion of RN staffing.

## Discussions

To the best of our knowledge, this is the first study designed to examine the longitudinal associations between nurse staffing and patient outcomes at both trend and seasonal levels for US hospitals. Using a grouping and aggregation approach, we were able to capture significant associations of staffing and patient outcomes at both trend and seasonal levels while including units with various missing data structures into analyses.

### Key findings

#### Unit-level random variation and its impact

The large within-unit variability (noise) may too large for studies on patient outcomes like falls and pressure ulcers to capture meaningful associations (signal). Due to such variability, seasonality was undetectable in fall or pressure ulcers rates at single unit level; with all units included, seasonal associations between staffing and falls and pressure ulcers were undetectable when we used hierarchical generalized linear model to conduct unit-level analyses (results not shown). It is possible that some longitudinal studies at the unit or hospital level, such as those that failed to find a beneficial impact of California’s nurse staffing legislation [5, 6], were not able to detect the impact of changes in staffing on patient outcomes because of large within-unit variability in the outcomes. On the other hand, a single hospital or unit may not immediately see better results in patient outcomes after implementing quality-improvement strategies for the same reason, especially when the outcomes are measured at the monthly or quarterly level.

#### Trend and seasonal associations

The trend-level inverse associations between changes in nurse staffing (total HPPD and RN skill-mix) and all four outcomes are consistent with the fact that RN staffing increased and rates of adverse outcomes decreased from 2004 to 2012 [4, 11, 16, 17]. The inverse associations between RN skill-mix and fall and pressures ulcer rates at trend level remained significant with control for total HPPD, suggesting that the composition of nursing staff matters. Annual non-RN staffing level was relatively stable except for a sudden drop around 2009 [4]. This drop was consistent across groups in this study (see Additional file 2: Figure S2, which demonstrates time trends in staffing variables). At about the same time, the rate of pressure ulcers of stage III or above also dropped consistently across groups (Fig. 3). The possible connection between these two sudden changes needs further examination in the future.

The 2008 CMS rule change may provide some explanation for these study results. Starting in 2008, CMS required hospitals to track “present on admission (POA)” conditions for falls and pressure ulcers and other outcomes and stopped reimbursing hospitals for the extra costs for treating non-POA conditions. It is expected that hospitals would initiate mechanisms to better track these POA conditions and take actions to prevent falls and pressure ulcers in order to receive full reimbursement from CMS. The sudden decrease in the rate of pressure ulcers of stage III or above around 2009 suggests that some hospitals may have succeeded in preventing more stage I/II pressure ulcers from deteriorating into more costly stages. Although non-RN HPPD dropped around 2008, RN HPPD kept increasing thus the total HPPD kept increasing. It is possible that some hospitals reduced non-RNs to increase RNs in response to the CMS policy change.

Interpretations of the seasonal associations of staffing and patient outcomes are less straightforward. Total HPPD overall increased from Quarter 1 to Quarter 4 in a year, whereas RN skill-mix tended to decrease from Quarter 1 to Quarter 2 and increase from Quarter 3 to Quarter 4 (see Additional file 3: Figure S3, which demonstrates seasonal patterns in staffing variables). RN skill-mix varied across seasons because RN and non-RN staffing changed differently across seasons. RN HPPD kept increasing from year to year and from quarter to quarter except that it tended to plummet in Quarter 1 of each year when patient volume tended to peak [11]. Non-RN HPPD was overall stable at trend level (except for the sudden drop around 2009) but changed regularly at seasonal level, likely due to the seasonal change in patient volume. Quarter 1 and Quarter 4 in a year were more likely to have lower staffing, but RN skill-mix tended to be higher in these quarters. Thus, it is not surprising to see positive association of RN skill-mix with falls and pressure ulcers at seasonal level.

Seasonal nursing shortage could have contributed to seasonality in patient outcomes. In winter, staffing level tended to be lower than other seasons even though the total staffing hours were higher because the increases in patient volumes were much greater [11]. Winter peak in overall or disease-specific hospital admissions has been reported in many other countries, such as Bangladesh [22], Canada [23], Denmark [24], Israel [25], Japan [26], and United Kingdom [27] etc. Meanwhile, nursing shortage was considered a global issue that International Council of Nurses (ICN) and other international nursing institutes initiated a global review in 2004 to identify the policy and practice issues and solutions [28]. What found in this study based on US hospitals is consistent with the global situation.

### Limitations

This study is limited from several aspects, for which the conclusions may be drawn from the findings are restricted. More rigorous future studies are needed to confirm the findings.

The analysis approach was limited to the existing database. No patient information is available for patient-level analysis as well as reducing unexplained variation (noise); data aggregation across units reduces noise but forsakes the direct association between staffing and outcomes at unit level. Furthermore, inconsistent reporting patterns across units led to data aggregation based on data missing patterns that has little meaningful interpretations.

The association between nurse staffing and patient outcomes found in time trend cannot tells us if or how much of the improvements in patient outcomes was due to the increases in nurse staffing. Various factors may have contributed to the improvement in patient outcomes, such as changes in nursing practice, new technology, and other unit- or hospital-level quality improvement efforts. There was no control for these potential confounders in our study. If researchers plan to conduct an observational longitudinal study in the future, it is not clear how these factors can be effectively controlled given the difficulty of identifying and measuring these factors all across units and hospitals. This remains a challenge for future study.

Seasonal associations, by contrast, are unlikely to be substantially confounded with intentional improvement in patient care from other aspects. However, other factors may change seasonally and impact patient outcomes. When patient volume increases, the availability of other ancillary resources and supplies may also be reduced, thus increasing the risk of falls and pressure ulcers. Pressure ulcers are more likely to happen in winter maybe because of the relatively dry skin in winter [22] or winter illnesses that may be related to patients less mobile, such as older patients with strokes or cardiovascular diseases [22, 25, 29, 30].

Aggregation analysis may hide some important information. Our aggregation-based analyses prevented us from differentiating staffing-outcome associations by hospital and other unit-level characteristics, such as hospital Magnet status, hospital teaching status, hospital size, and unit type. Besides using aggregation to average out the variations, another approach is to explain part of the variation, such as incorporating extensive patient information to conduct patient level analyses. How much the inclusion of such variables would reduce the variation, and whether the reduction is enough to reveal the association of staffing and patient outcomes are unknown.

To form large groups for aggregation analyses, some quarterly observations were deleted. We do not expect deleting these observations to fundamentally change the results, but it may have brought biases. Multiple imputations for missing staffing variables and patient outcomes at unit-level may be considered in the future.

## Conclusion

This study is unique in finding that changes in nurse staffing were inversely associated with changes in the rates of falls and pressure ulcers at both the time trend and the seasonal levels. No causal inference about staffing and patient outcomes can be made without control for improvements in quality of patient care from other aspects or changes in patient population over time, or other seasonal factors that may have influenced patient outcomes at the seasonal level. We hypothesize that increased staffing levels have contributed to reducing falls and pressure ulcers in recent years, and that fluctuations in nurse staffing due to seasonal changes in patient volume have immediate impact on risk for falls and pressure ulcers. More rigorous studies are needed to test these hypotheses. Besides increasing nurse staffing level, improving nursing education and working environment to meet the increasing nursing needs, hospitals also need more flexible seasonal nursing models. With big data, hospitals may build more efficient nursing prediction and management system combining environmental factors, patient characteristics, process data, and national or international resources.

Our findings also suggest that large temporal variations in patient outcomes may prevent us from finding meaningful longitudinal associations. The potential impact of such variations should be taken into consideration in future research. Patient level analysis with control for patient characteristics, disease types, and severity may reduce such variation to reveal more direct longitudinal associations of nurse staffing and patient outcomes.

## Additional files

Additional file 1: Figure S1. Flowchart of data preparation for final analyses. (EPS 2329 kb)
Additional file 2: Figure S2. The annual means of changes from baseline in four nursing variables aggregated over units within 42 groups of falls data: nursing hours per patient day provided by registered nurses (RN HPPD) and non-registered nurses (non-RN HPPD), total HPPD (RN HPPD + non-RN HPPD), and percent of nursing hours provided by RNs (RN skill-mix). The thick line in each plot is for the group of 1240 units with all 36 quarters of data in 2004–2012. Plots based on data for pressure ulcers show similar patterns. Each vertical dash line denotes a year. (EPS 401 kb)
Additional file 3: Figure S3. The seasonal variation in four nursing variables aggregated over units within 42 groups in the data for falls: nursing hours per patient day provided by registered nurses (RN HPPD) and non-registered nurses (non-RN HPPD), total HPPD (RN HPPD + non-RN HPPD), and percent of nursing hours provided by RNs (RN skill-mix). The thick line in each plot is for the group of 1240 units with all 36 quarters of data in 2004–2012. Plots based on data for pressure ulcers show similar patterns. Each vertical dash line denotes Quarter 4 of a year. (EPS 456 kb)

## Abbreviations

ANA: The American Nurses Association
CMS: The Centers for Medicare & Medicaid Services
HPPD: Nursing hours per patient days
NDNQI: National Database of Nursing Quality Indicators® (NDNQI®)
POA: Present on admission
RN: Registered nurses
